# Supplementary material for: Mesenchymal Stem/ Stromal Cells metabolomic and bioactive factors profiles: A comparative analysis on the umbilical cord and dental pulp derived Stem/ Stromal Cells secretome
Source: PLoS One. 2019 Nov 27;14(11):e0221378. doi: 10.1371/journal.pone.0221378 (PMC6881058; doi:10.1371/journal.pone.0221378)
Supplement: S1 Table — Calculated from 1H-NMR spectra of unconditioned (Plain Medium), and UC-MSCs and DPSCs conditioned media, after 24 and 48 hours. Significance of the results is indicated according to P values with one, two, three or four of the symbols (*) corresponding to 0.01≤P<0.05; 0.001≤P< 0.01; 0.0001≤P<0.001 and P<0.0001, respectively; ns, not significant. (DOCX) [file pone.0221378.s001.docx]

**Supporting Information:**

**S1 Table:** **Statistically significant differences in Metabolites concentration (µM).** Calculated from ^1^H-NMR spectra of unconditioned (Plain Medium), and UC-MSCs and DPSCs conditioned media, after 24 and 48 hours. Significance of the results is indicated according to P values with one, two, three or four of the symbols (*) corresponding to 0.01≤P<0.05; 0.001≤P< 0.01; 0.0001≤P<0.001 and P<0.0001, respectively; ns, not significant.

| ***Metabolite*** | ***Group*** | ***Chem. Shift (ppm)*** | ***Statistically Significant Differences in Metabolite Concentration (μM)*** | | | | | | | | | | |  |
| --- | --- | --- | --- | --- | --- | --- | --- | --- | --- | --- | --- | --- | --- | --- |
|  |  |  | ***Plain Medium*** | | | | ***UC-MSCs 24h*** | | | ***UC-MSCs 48h*** | | ***DPSCs 24h*** | |  |
|  |  |  | ***vs UC 24h*** | ***vs UC 48h*** | ***vs DP 24h*** | ***vs DP 48h*** | ***vs UC 48h*** | ***vs DP 24h*** | ***vs DP 48h*** | ***vs DP 24h*** | ***vs DP 48h*** | | ***vs DP 48h*** | |
| **Acetate** | CH_3_ | 1.93 | ** | * | ns | ns | ns | ** | *** | ** | ** | | ns | |
| **Alanine** | CH_3_ | 1.48 | **** | **** | **** | **** | **** | **** | **** | **** | **** | | **** | |
| **Choline** | N(CH_3_)_3_ | 3.21 | ** | * | * | * | ns | ns | ns | ns | ns | | ns | |
| **Ethanol** | CH_3_ | 1.19 | **** | **** | **** | **** | **** | ns | **** | **** | **** | | **** | |
| **Formate** | CH | 8.46 | ns | * | ns | ns | ns | ns | ns | ns | ns | | ns | |
| **GlutaMAX I (L-Ala)** | CH_3_ | 1.42 | * | **** | ns | **** | **** | ns | **** | **** | *** | | **** | |
| **GlutaMAX II (L-Glu)** | CH_2_ | 2.34 | ns | **** | ns | **** | **** | ns | **** | **** | ns | | **** | |
| **Glutamine** | CH_2_ | 2.45 | **** | **** | * | **** | ** | **** | ns | **** | ns | | **** | |
| **Lactate** | CH | 4.12 | **** | **** | **** | **** | ns | * | * | *** | ns | | *** | |
| **Nicotinamide** | CH | 8.94 | ns | ns | ns | ns | ns | ns | ns | ns | ns | | ns | |
| **Pyruvate** | CH_3_ | 2.37 | ns | ns | ns | ns | * | ns | ns | ns | ns | | ns | |
| **Tyrosine** | 2CH | 7.19 | **** | ** | * | ** | ** | *** | *** | ns | ns | | ns | |
| **α-Glucose** | CH | 5.24 | **** | ns | ns | * | **** | **** | **** | ns | ns | | ns | |
| **β-Glucose** | CH | 4.65 | **** | ns | ns | ns | *** | **** | **** | * | ns | | ns | |
