# Supplementary material for: Mesenchymal Stem/ Stromal Cells metabolomic and bioactive factors profiles: A comparative analysis on the umbilical cord and dental pulp derived Stem/ Stromal Cells secretome
Source: PLoS One. 2019 Nov 27;14(11):e0221378. doi: 10.1371/journal.pone.0221378 (PMC6881058; doi:10.1371/journal.pone.0221378)
Supplement: S2 Table — Calculated from Multiplexing LASER Bead Analysis of UC-MSCs and DPSCs Conditioned Media after 24 and 48 hours of conditioning. Significance of the results is indicated according to P values with one, two, three or four of the symbols (*) corresponding to 0.01≤P<0.05; 0.001≤P<0.01; 0.0001≤P<0.001 and P<0.0001, respectively; ns, not significant. (DOCX) [file pone.0221378.s002.docx]

**Supporting Information:**

**S2 Table:** **Statistically significant differences on detected Bioactive factors (pg/mL).** Calculated from Multiplexing LASER Bead Analysis of UC-MSCs and DPSCs Conditioned Media after 24 and 48 hours of conditioning. Significance of the results is indicated according to P values with one, two, three or four of the symbols (*) corresponding to 0.01≤P<0.05; 0.001≤P<0.01; 0.0001≤P<0.001 and P<0.0001, respectively; ns, not significant.

| ***Bioactive Factor*** | ***Statistical significance*** | | | |
| --- | --- | --- | --- | --- |
|  | ***UC 24h vs*** | ***DP 24hvs*** | ***UC 24h vs*** | ***UC 48h vs*** |
|  | ***UC 48h*** | ***DP 48h*** | ***DP 24h*** | ***DP 48h*** |
| **Eotaxin-1** | **** | ns | ns | **** |
| **FGF-2** | **** | ns | ns | **** |
| **Follistatin** | - | ns | ns | - |
| **Fractalkine** | **** | ns | ns | **** |
| **GRO pan** | * | ** | ns | ns |
| **HGF** | ns | - | - | - |
| **IFNα2** | **** | ns | ns | **** |
| **IL-1α** | *** | - | - | *** |
| **IL-6** | **** | ns | ns | **** |
| **IL-8** | ns | ns | ns | ns |
| **MCP-1** | **** | - | - | - |
| **MCP-3** | **** | ns | ** | **** |
| **PDGF-BB** | **** | ns | ns | **** |
| **RANTES** | **** | - | - | **** |
| **TGF-β1** | *** | ns | ns | *** |
| **TGF-β2** | - | - | - | - |
| **VEGF-A** | **** | ns | ns | **** |
| **VEGF-C** | **** | ns | ns | **** |
