# Supplementary material for: Mesenchymal Stem/ Stromal Cells metabolomic and bioactive factors profiles: A comparative analysis on the umbilical cord and dental pulp derived Stem/ Stromal Cells secretome
Source: PLoS One. 2019 Nov 27;14(11):e0221378. doi: 10.1371/journal.pone.0221378 (PMC6881058; doi:10.1371/journal.pone.0221378)
Supplement: S4 Table — After 48 hours of expansion in Complete, Control or CMs supplemented media, according to P values with one, two, three or four of the symbols (*) corresponding to 0.01≤P<0.05; 0.001≤P<0.01; 0.0001≤P<0.001 and P<0.0001, respectively; ns, not significant. (DOCX) [file pone.0221378.s004.docx]

**Supporting Information:**

**S4 Table:** **Significance of the results of the Apoptosis (Annexin-V/ PI) assay of UVECs.** After 48 hours of expansion in Complete, Control or CMs supplemented media, according to P values with one, two, three or four of the symbols (*) corresponding to 0.01≤P<0.05; 0.001≤P<0.01; 0.0001≤P<0.001 and P<0.0001, respectively; ns, not significant.

| ***Viable Cells*** | **Complete medium** | **Control** | **UC-MSCs CM** | **DPSCs CM** |  |  | ***Early***  ***Apoptosis*** | **Complete medium** | **Control** | **UC-MSCs CM** | **DPSCs CM** |
| --- | --- | --- | --- | --- | --- | --- | --- | --- | --- | --- | --- |
|  |  |  |  |  |  |  |  |  |  |  |  |
| **Complete media** |  | **** | ns | ns |  |  | **Complete media** |  | **** | * | ns |
| **Control** |  |  | **** | **** |  |  | **Control** |  |  | **** | **** |
| **UC-MSCs CM** |  |  |  | * |  |  | **UC-MSCs CM** |  |  |  | * |
| **DPSCs CM** |  |  |  |  |  |  | **DPSCs CM** |  |  |  |  |
|  |  |  |  |  |  |  |  |  |  |  |  |
| ***Late***  ***Apoptosis*** | **Complete medium** | **Control** | **UC-MSCs CM** | **DPSCs CM** |  |  | ***Dead Cells*** | **Complete medium** | **Control** | **UC-MSCs CM** | **DPSCs CM** |
|  |  |  |  |  |  |  |  |  |  |  |  |
| **Complete media** |  | **** | ns | ns |  |  | **Complete media** |  | ns | ns | ns |
| **Control** |  |  | ** | ** |  |  | **Control** |  |  | ns | ns |
| **UC-MSCs CM** |  |  |  | ns |  |  | **UC-MSCs CM** |  |  |  | * |
| **DPSCs CM** |  |  |  |  |  |  | **DPSCs CM** |  |  |  |  |
